# Pathophysiology and pathogenesis of circadian rhythm sleep disorders

**DOI:** 10.1186/1880-6805-31-7

**Published:** 2012-03-13

**Authors:** Akiko Hida, Shingo Kitamura, Kazuo Mishima

**Affiliations:** 1 Department of Psychophysiology, National Institute of Mental Health, National Center of Neurology & Psychiatry, 4-1-1 Ogawa-Higashi, Kodaira, Tokyo 187-8553, Japan

**Keywords:** circadian, sleep, surrogate measurement, clock gene expression, biopsy sample

## Abstract

Metabolic, physiological and behavioral processes exhibit 24-hour rhythms in most organisms, including humans. These rhythms are driven by a system of self-sustained clocks and are entrained by environmental cues such as light-dark cycles as well as food intake. In mammals, the circadian clock system is hierarchically organized such that the master clock in the suprachiasmatic nuclei of the hypothalamus integrates environmental information and synchronizes the phase of oscillators in peripheral tissues. The transcription and translation feedback loops of multiple clock genes are involved in the molecular mechanism of the circadian system. Disturbed circadian rhythms are known to be closely related to many diseases, including sleep disorders. Advanced sleep phase type, delayed sleep phase type and nonentrained type of circadian rhythm sleep disorders (CRSDs) are thought to result from disorganization of the circadian system. Evaluation of circadian phenotypes is indispensable to understanding the pathophysiology of CRSD. It is laborious and costly to assess an individual's circadian properties precisely, however, because the subject is usually required to stay in a laboratory environment free from external cues and masking effects for a minimum of several weeks. More convenient measurements of circadian rhythms are therefore needed to reduce patients' burden. In this review, we discuss the pathophysiology and pathogenesis of CRSD as well as surrogate measurements for assessing an individual's circadian phenotype.

## Mammalian circadian clock system

**Figure 1:**
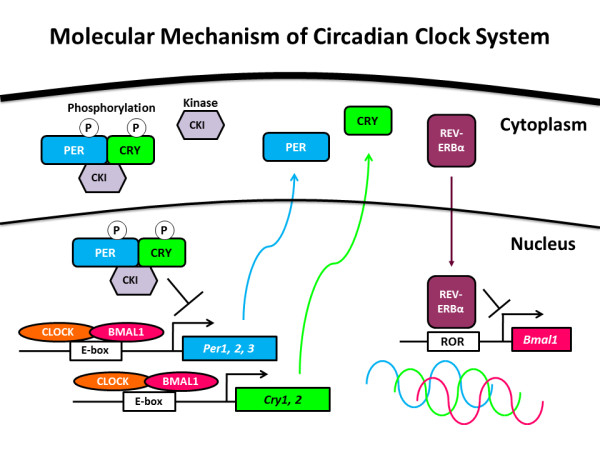
**Molecular mechanism of circadian clock system**.

The circadian clock system regulates daily rhythms of physiology and behavior, such as the sleep-wake cycle and hormonal secretion, body temperature and mood [1]. These rhythms are entrained by environmental cues, light-dark (LD) cycles and food intake. In mammals, the master clock in the suprachiasmatic nuclei (SCN) of the hypothalamus incorporates environmental information and coordinates the phase of oscillators in peripheral cells, tissues and organs [2,3]. Light is one of the most potent environmental cues that enable the organisms to adapt to the 24-hour environmental LD cycle. Photic signals are delivered from the eye to the SCN via the retinohypothalamic tract, thereby mediating the entrainment of the circadian clock system [4]. The circadian clock system involves transcription-translation negative feedback loops of multiple clock genes and posttranscriptional modification and degradation of clock proteins [4-6] (Figure 1). The basic helix-loop-helix and Per-Arnt-Sim transcription factors CLOCK and BMAL1 form heterodimers and activate transcription of *Period 1 *(*Per1*), *Per2*, *Per3*, *Cryptochrome 1 *(*Cry1*), *Cry2 *and *retinoid-related orphan receptor α *(*Rorα*), *Rorβ*, *Rorγ*, *Rev-Erbα *and *Rev-Erbβ *by binding to E-box motifs on their promoter regions. PER and CRY proteins gradually accumulate in the cytoplasm and phosphorylation of PER and CRY occurs with casein kinase Iδ (CKIδ) and CKIε. PER, CRY and CKI proteins form complexes that translocate to the nucleus and interact with CLOCK-BMAL1 heterodimers, thereby inhibiting transcription of the *Per*, *Cry*, *Ror *and *Rev-Erb *genes. Meanwhile, *Bmal1 *transcription is regulated positively by retinoid-related orphan receptor (ROR) and negatively by REV-ERB via the ROR element (RORE) motif on the *Bmal1 *promoter.

## Circadian rhythm sleep disorders

A two-process model is a major model of sleep regulation. Two components, homeostatic drive and circadian drive, interact with each other and regulate the sleep-wake cycle [7]. The sleep-wake cycle is controlled by sleep homeostasis. The desire to sleep increases gradually with extended wakefulness and decreases during sleep. Additionally, sleep and wakefulness occur in turn, and the timing of their occurrence is controlled by the circadian clock system. Circadian rhythm sleep disorders (CRSDs) are defined by a persistently or recurrently disturbed sleep pattern. CRSD is attributed etiologically to alterations of the circadian timekeeping system and/or a misalignment between endogenous circadian rhythm and exogenous factors that affect sleep timing [8]. The intrinsic circadian period (τ, the free-running period of circadian rhythms in the absence of external cues) is considered to be a critical factor in the pathophysiology of CRSD [9,10].

### Familial advanced sleep phase type

Familial advanced sleep phase type (FASPT) is an autosomal dominant genetic disease characterized by extremely early involuntary sleep timing. A missense mutation in the *PER2 *gene has been identified in a large pedigree with FASPT. This mutation caused a change from serine to glycine at amino acid 662 (S662G) located in the CKIε binding domain of the PER2 protein and resulted in decreased PER2 phosphorylation [11]. Transgenic mice carrying the mutant S662G *PER2 *gene showed a shorter free-running period, τ [12]. In addition, a missense mutation in the *CKIδ *gene was found in another FASPT pedigree. The substitution of threonine with alanine at amino acid 44 of CKIδ reduced enzymatic activity of CKIδ, leading to decreased phosphorylation level of PER2, a target of CKI [13]. The CKIδ T44A mutation shortened τ, as well as the PER2 S662G mutation, in mice. It was previously proposed that decreased phosphorylation of PER2 stabilizes the PER2 protein, thereby enhancing nuclear accumulation of PER2 and leading to a shorter circadian period. Recent studies, however, have shown that decreased PER2 phosphorylation enhances destabilization of PER2 by increasing turnover and degradation of PER2 [14,15]. These findings suggest that the shortening of τ observed in the FASPT models results from enhanced turnover of nuclear PER2 caused either by increased degradation or by reduced nuclear retention. FASPT patients have been reported to have a shorter period of physiological rhythms [16]. Several studies have indicated that the phosphorylation status of circadian clock proteins plays a critical role in regulating circadian periods [17,18]. Altered τ seems to contribute to the pathogenesis of CRSD.

### Delayed sleep phase type

Delayed sleep phase type (DSPT) is characterized by the inability to fall asleep and awaken at a desired time, leading to significantly later sleep onset and wake times. The pathophysiology of DSPT is attributed to longer τ, misaligned phase relationship between endogenous clock and sleep-wake cycles, reduced photic entrainment and/or altered sleep homeostasis. The human *PER3 *gene has multiple missense polymorphisms that cause amino acid substitution and a variable number tandem repeat (VNTR) polymorphism that encodes either four or five copies of eighteen amino acids [19]. Association studies have shown that the longer allele (five copies) in *PER3 *VNTR polymorphism (*PER3*^5^) is associated with extreme morning preference and that the shorter allele (four copies) is associated with extreme evening preference and DSPT [20]. *PER3*^5 ^homozygotes have been reported to show increased slow-wave sleep in non-rapid eye movement sleep and θ/α activity during wakefulness compared to homozygotes for *PER3*^4 ^[21]. These results suggest that the *PER3 *polymorphism may be linked to homeostatic regulation of human sleep. The mouse *Per3 *gene was thought to be dispensable for circadian rhythm, as PER3-deficient mice did not show altered expression patterns of circadian clock genes in the SCN or altered behavioral rhythm [22]. However, PER3-deficient mice have recently been reported to have a shorter τ and advanced phase of *Per1 *rhythm in peripheral tissues compared to wild-type mice. The results suggest that *Per3 *may play a role in regulating circadian rhythms in the periphery [23]. Another group has found that PER3-deficient mice had a lower light sensitivity and suggested that *Per3 *may be involved in the light input pathway [24]. These findings imply that the function of the *PER3 *gene may contribute to the interaction between the circadian system and sleep homeostasis.

### Nonentrained type (free-running type)

Nonentrained type is characterized by sleep timing that occurs with a 30-minute to 1-hour delay each day. Nonentrained sleep-wake patterns are usually observed in totally blind people [25-27], whereas the nonentrained patterns are rarely observed in sighted people. It is likely that blind individuals have free-running rhythms due to the loss of photic reception (photic entrainment). Because the τ in humans is not extensively longer than 24 hours (average τ = 24.18 hours) [28] and sighted people are capable of perceiving photic signals, impaired photic entrainment as well as prolonged τ may underlie the pathophysiology of sighted patients with the nonentrained type.

## Evaluation of individual circadian phenotypes

FASPT, DSPT and nonentrained type of CRSDs are thought to result from malfunction and/or maladaptation of the circadian system. Evaluation of an individual's circadian phenotype is indispensable to understanding the pathophysiology of CRSD. Individual subjects are required to stay in a laboratory environment free from external cues during a couple of weeks' time to assess circadian rhythms precisely [28-30]. First, rhythmic characteristics of physiological functions (core body temperature, plasma melatonin and plasma cortisol levels) are measured to estimate individual circadian phases. Blood samples are collected over a 40-hour period under constant routine (CR) conditions where masking effects (for example, physical movement, food intake, ambient temperature and light intensity) are minimized (first CR). Next, patients undergo a 28-hour forced desynchrony (FD) protocol (9.33-hour sleep and 18.67-hour wake cycle) followed by a 40-hour CR (second CR). Individual circadian phases are assessed again during the second CR. The intrinsic circadian period, τ, is determined by the difference in circadian phase between the first and second CRs. As described herein, the CR and FD protocols are laborious and costly to perform in a clinical setting. More convenient measurements of circadian phenotypes are required to reduce the patients' burden.

## Surrogate measurements for assessing circadian phenotypes

**Figure 2:**
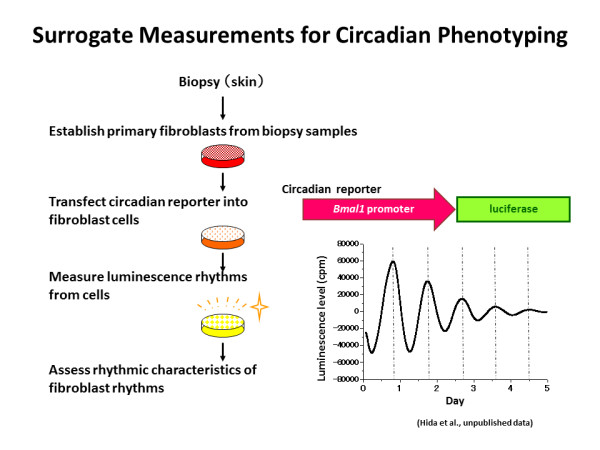
**Surrogate measurements for circadian phenotypes**.

Most cells in peripheral tissues as well as cells in the SCN are equipped with circadian clock components. Brown *et al*. developed a lentiviral luminescence assay system using biopsy samples to measure individual circadian rhythms in fibroblasts [31]. Primary cells derived from skin biopsy samples were introduced with a circadian reporter: the *Bmal1 *promoter-driven luciferase gene (*Bmal1*-*luc*). The luciferase activity under the control of the *Bmal1 *promoter showed robust daily rhythms in individual primary fibroblast cells. *Bmal1*-*luc *rhythms were monitored for several days, and rhythmic characteristics of the luminescence rhythms were evaluated. Independently, we measured clock gene expression in primary fibroblast cells established from individual skin biopsies and observed robust *Bmal1*-*luc *rhythms (Figure 2). Brown *et al*. found that extreme morning types had shorter periods of fibroblast rhythms compared to extreme evening types [32]. Furthermore, they compared the period length of fibroblast rhythms with that of physiological rhythms in the same subjects and observed a significant correlation between the two rhythms. However, they did not observe long fibroblast periods in blind subjects, who had significantly longer physiological rhythms than sighted subjects [33]. The prolonged physiological period observed in the blind subjects may be caused by their previous sleep-wake cycles under constant darkness. The unaltered fibroblast period may be attributed to experimental conditions. Although the reason for this discrepancy is not yet fully understood and further studies are required, surrogate measurements using fibroblast cells should be a powerful tool for assessing individual circadian properties.

## Conclusions

Evaluation of circadian phenotypes is indispensable to understanding the pathophysiology and pathogenesis of CRSD. Because conventional protocols for examining individual circadian characteristics are laborious and costly, more convenient measurement methods are required in the clinical setting. The circadian reporter *Bmal1*-*luc *showed robust daily rhythms in primary fibroblast cells derived from individual skin biopsies. The fibroblast rhythms are associated with chronotypes (morningness vs eveningness preference) and physiological rhythms. Surrogate measurements using fibroblast cells would be a powerful tool for the assessment of individual circadian properties and could lead to providing personalized medicine for CRSD.

## Competing interests

The authors declare that they have no competing interests.

## Authors' contributions

AH wrote the manuscript and performed circadian phenotyping. SK and KM edited the manuscript. All authors read and approved the final manuscript.
